# A Rat Model of Alzheimer’s Disease Based on Abeta_42_ and Pro-oxidative Substances Exhibits Cognitive Deficit and Alterations in Glutamatergic and Cholinergic Neurotransmitter Systems

**DOI:** 10.3389/fnagi.2016.00083

**Published:** 2016-04-20

**Authors:** Tomas Petrasek, Martina Skurlova, Kristyna Maleninska, Iveta Vojtechova, Zdena Kristofikova, Hana Matuskova, Jana Sirova, Karel Vales, Daniela Ripova, Ales Stuchlik

**Affiliations:** ^1^Department of Neurophysiology of Memory, Institute of Physiology of the Czech Academy of SciencesPrague, Czech Republic; ^2^National Institute of Mental HealthKlecany, Czech Republic

**Keywords:** animal model, Alzheimer’s disease, sporadic AD, learning and memory, cognition, neurochemistry of the acetylcholine system, hippocampus

## Abstract

Alzheimer’s disease (AD) is one of the most serious human, medical, and socioeconomic burdens. Here we tested the hypothesis that a rat model of AD (Samaritan; Taconic Pharmaceuticals, USA) based on the application of amyloid beta_42_ (Abeta_42_) and the pro-oxidative substances ferrous sulfate heptahydrate and L-buthionine-(S, R)-sulfoximine, will exhibit cognitive deficits and disruption of the glutamatergic and cholinergic systems in the brain. Behavioral methods included the Morris water maze (MWM; long-term memory version) and the active allothetic place avoidance (AAPA) task (acquisition and reversal), testing spatial memory and different aspects of hippocampal function. Neurochemical methods included testing of the NR1/NR2A/NR2B subunits of NMDA receptors in the frontal cortex and CHT1 transporters in the hippocampus, in both cases in the right and left hemisphere separately. Our results show that Samaritan rats^™^ exhibit marked impairment in both the MWM and active place avoidance tasks, suggesting a deficit of spatial learning and memory. Moreover, Samaritan rats exhibited significant changes in NR2A expression and CHT1 activity compared to controls rats, mimicking the situation in patients with early stage AD. Taken together, our results corroborate the hypothesis that Samaritan rats are a promising model of AD in its early stages.

## Introduction

Alzheimer’s disease (AD) is a serious neuropsychiatric disorder, invariably resulting in the death of the patient, preceded by a slow and excruciating deterioration of memory, cognitive abilities and personality, which constitutes a serious burden not only for patients but also for their relatives and the whole society. With the ageing population in many countries, the number of people suffering from AD will increase enormously. The exact etiology of the disease is not known, although it presents with extracellular plaques of amyloid beta (Abeta) peptides and intracellular tangles of protein tau (Reiman, 2014). Both these factors are suspected to play an important role in disease progression (Spires-Jones and Hyman, 2014). Apart from protein accumulations, AD is accompanied by neuroinflammation, oxidative stress, and neurodegeneration of the cholinergic, noradrenergic and serotonergic projections (Wenk, 2003). Many scientists today are not convinced that the pathological accumulation of proteins is the primary cause of the disease process, and consider neuroinflammation and disrupted axonal transport as possible factors that may be present prior to the clinical onset of the disorder (Schuitemaker et al., 2009; Ye et al., 2012). Multiple changes reaching beyond the simple description given above have been detected in AD brain neurochemistry (e.g., Cai and Ratka, 2012). These changes can be studied primarily in animal models and *post mortem* studies, since the living human brain is barely accessible to biochemical examination.

There are two basic forms of AD: familial and sporadic. The familial form is very rare and is related to inherited gene mutations (Rossor et al., 1993). The sporadic form represents a vast majority of AD cases (Piaceri et al., 2013) and typically occurs at more advanced age. There are reports that this type may start in middle age as well, although this is not very common (Reiman, 2014). A vast majority of transgenic models of AD carry various mutations in Abeta, tau or presenilin (Do Carmo and Cuello, 2013). In this respect, these models are closer to the familial, rather than sporadic, form of AD. However, valid models of sporadic AD, which may not necessarily involve gene mutations, are of high importance for basic and applied research focused on AD (Lecanu and Papadopoulos, 2013).

Beside massive neurodegeneration and neurochemical and anatomical changes in the brain, patients with AD exhibit severe learning and memory deficits. These include disorientation and other impairments affecting the cognitive domain (Reiman, 2014). Therefore, appropriately sensitive behavioral testing of these models coupled to detailed examinations of brain biochemistry is very important.

For the present study we used a rat model of AD based on the chronic (28 days) intracerebroventricular application of Abeta_42_ and the pro-oxidative substances ferrous sulfate heptahydrate and L-buthionine-(S, R)-sulfoximine (the Samaritan Alzheimer’s Rat Model; Taconic Pharmaceuticals, USA, described in Lecanu et al., 2006). We must note that this model is mainly focused on mimicking the symptoms of the disease, and is therefore not suitable for elucidating its etiology. A previous study (Lecanu et al., 2006) documented impairments of working memory and typical neuropathological changes in this model. We attempted to characterize the model further, using two independent spatial cognitive tasks focused mostly on hippocampal function together with a neurochemical analysis of the hippocampus.

Our working hypothesis was that the Samaritan rat model of sporadic AD would present cognitive deficits in spatial tasks and alterations in brain glutamatergic and cholinergic neurotransmitter systems.

The behavioral tasks we employed, the Morris water maze (MWM) and the active allothetic place avoidance task (AAPA), place very different demands on hippocampal functions. Solving the MWM requires storing precise representation of spatial relationships in order to locate a small, hidden goal. In the AAPA, on the other hand, the major hippocampus-dependent task involves organizing the spatial information into two conflicting frames, and selection of the relevant one. Impairments of hippocampal function therefore impact preferentially memory retrieval in the MWM, whereas in the AAPA, new learning is disrupted before retrieval (Kubík and Fenton, 2005). Therefore, we were interested in the possibility of differential impacts of AD-related cognitive decline on performance in these tests.

## Materials and Methods

### Animals

Young adult male Long-Evans rats were obtained from Taconic Pharmaceuticals, USA (with the patented commercial name Samaritan rat^™^; see Lecanu et al., 2006), where they underwent the following procedure prior to delivery. The experimental animals received an infusion of Abeta_42_ (15 μM) and two pro-oxidative substances, ferrous sulfate heptahydrate (1 mM) and L-buthionine-(S, R)-sulfoximine (12 mM), dissolved in artificial cerebrospinal fluid. The solution was applied chronically (4 weeks at a rate of 2.5 μl/h) via an osmotic pump connected to a permanent cannula inserted into the left cerebral ventricle, to induce AD-like brain pathology. This procedure was described in detail by Lecanu et al. (2006). Control rats were sham-treated (infused by the solvent without active substances). Treatments started at the age of 7 postnatal weeks, and lasted for 4 weeks.

At the age of 8 weeks, the animals were transported from Taconic Pharmaceuticals to the Institute of Physiology, Academy of Sciences of the Czech Republic, where they were allowed a 2-week acclimatization period. Animals were housed in an accredited animal room with constant humidity (50%), temperature (22 ± 1°C) and a regular light-dark cycle (lights on between 6:00 and 18:00). Animals from both groups were tested in an alternating order, to exclude bias from potential circadian changes in performance.

All experiments were approved by the local Committee for Animal Protection and complied with the Animal Protection Act of the Czech Republic, EU directive (2010/63/EC). Access to water and standard rat food was ad libitum.

From an initial total of 21 animals, two died, and one of the animals from the experimental group had to be excluded because of non-standard behavior (the animal was obviously anxious, aggressive when manipulated and attempted to escape from the testing apparatus). Therefore, nine Samaritan and ten control rats, aged 11–22 weeks, were used in the study.

### Study Design

After the acclimatization period at the Institute of Physiology AS CR, animals were handled for 5 days (handling included habituation to human touch, holding and manipulation by the experimenters for 5–10 min per day) and then subjected to MWM testing for 6 days starting at the age of 11 weeks, and then tested in an AAPA test with reversal, starting at the age of 20 weeks. After completion of the behavioral studies, the rats were sacrificed at the age of 22 weeks and subsequent neurochemical analyses were conducted *post mortem*. Table 1 shows the timing of the most important experimental steps.

**Table 1:** **Experimental design**.

Week 7	Week 8–10	Week 11–12			Week 20–22			Week 22
Surgery (Taconic Pharmaceuticals, USA)	Arrival to the animal room, acclimation period, handling	Morris Water Maze			AAPA			Brain tissue sampling
		4 days acquisition	1 day probe trial	1 day visible platform	2 days habituation	5 days acquisition	5 days reversal		

### The Morris Water Maze (MWM)

The MWM is a classical test of precise place navigation and memory (Morris, 1981; Stuchlik et al., 2007a; Petrásek et al., 2014a), widely used in models of cognitive disorders. We used a reference memory protocol with four-day training, followed by a probe trial at Day 5 and visible platform testing at Day 6. The MVM (Morris, 1981; Stuchlik et al., 2007b; Petrásek et al., 2014a; reviewed in D’Hooge and De Deyn, 2001) consisted of a gray circular pool (180 cm in diameter) filled with water at a temperature of 21 ± 2°C to a depth of 35 cm. The water was rendered opaque by adding a small amount of non-toxic white paint (Primalex, PPG Deco; Czech Republic). The maze was located in a room providing an abundance of extra-maze cues. Swimming trajectories were monitored by an overhead camera connected to a digital tracking system and data acquisition program (Tracker, Biosignal Group, NY, USA). The maze contained a transparent plastic platform (10 cm in diameter) located in the center of the NE quadrant (the quadrants were labelled based on arbitrary compass directions) in acquisition sessions of the hidden-platform phase. In total, there were four daily sessions of hidden-platform testing in the MWM with the same platform position. The rats were released for six swims per session, separated by 15-min intervals, from different start locations, the sequence of which was changed pseudo-randomly for each daily session. Probe trial (60-s swims with the platform removed from the pool) was given in the fifth daily session to demonstrate the remembered platform position (one session, one swim).

In the visible platform testing (one daily session) the platform was raised above the surface and marked by a dark rim. The animals underwent eight swims in 15-min intervals, always being released from pseudorandomly chosen places around the periphery. The platform position was located in the SE. All MWM experiments were done between 18:00 and 23:00, i.e., during the dark phase of the day.

### Active Allothetic Place Avoidance (AAPA)

AAPA is a spatial task on a dry arena (Czéh et al., 2001), constituting prototype of a dynamic memory test (Stuchlik, 2014). Compared to the MWM, it places lower demands on precise spatial navigation, but on the other hand, it requires segregation of spatial frames, a skill that is considered equivalent to human cognitive coordination (Wesierska et al., 2005; for detailed conceptualization of this term see Phillips and Silverstein, 2003). We also included reversal training, sensitive to cognitive flexibility impairments (Petrásek et al., 2014a,b). The AAPA task is a test of both spatial abilities and executive functions, requiring animals to maintain two spatial representations and choose the relevant one, and is especially sensitive to subtle damages of hippocampal function (Kubík and Fenton, 2005; Stuchlik et al., 2013; Petrásek et al., 2014a).

The active place avoidance apparatus (Carousel; originally described by Bures et al., 1997; Fenton et al., 1998; reviewed in Stuchlik et al., 2013, 2014) was a smooth metallic arena (82 cm in diameter), enclosed with a transparent Plexiglas wall (for details of the apparatus and procedures see Stuchlik et al., 2007a; Stuchlik and Vales, 2008; Lobellova et al., 2013). At the beginning of each session, a rat was placed in the center of the arena, which rotated constantly at one revolution per minute. A 60-degree to-be-avoided sector was defined in the coordinate frame of the room by the computer-based tracking system (Tracker, Biosignal Group, NY, USA), which also recorded the positions of the rat and the arena (which were both marked by infrared LED diodes) at a sampling rate of 25 Hz. Each entrance into the sector lasting more than 300 ms was punished by mild electric footshocks (AC, 50 Hz, repeated every 1200 ms until the rat left the sector) delivered by the tracking system. The intensity of the shock was individualized for each rat (0, 3–0, 7 mA), to ensure an escape reaction while avoiding freezing caused by excessive pain. The shocks were administered through a cable attached to a harness on the back of the rat and connected to a conductive subcutaneous implant. The current was perceived by the rat at places of high-impedance contact between the paws and the grounded metallic floor. The trajectories were digitized and recorded on a PC, allowing off-line reconstruction and analysis of the animal’s trajectory (Track Analysis, Biosignal Group, NY, USA; Carousel Maze Manager, Bahník, 2014) both in the coordinate frame of the room and in the coordinate frame of the rotating arena.

Since the arena rotated, the rat had to move actively away from the sector in the direction opposite to arena rotation, otherwise it would be passively transported into the shock sector. For successful avoidance, the animal had to distinguish the distant room-frame cues, which could be used to locate and avoid the sector, from the irrelevant, arena-frame cues (i. e. scent marks), which moved relative to the sector position and were thus misleading.

Testing began with two habituation sessions, one on a stable arena, the other with rotation, to observe the spontaneous behavior of the animals and allow them to become accustomed to the testing procedure. Ten daily 20-min sessions of active place avoidance testing were conducted separated by 24-h inter-trial intervals. The initial five sessions were considered acquisition sessions, and were followed by five reversal sessions with the sector position shifted by 180 degrees. Carousel maze testing was conducted during daylight hours (10:00–18:00).

### Neurochemical Analysis of the Brain Tissue

Neurochemical analysis was focused on changes in the cortical glutamatergic and hippocampal cholinergic neurotransmitter systems, both involved in learning and memory processes. With respect to changes in the glutamatergic system in AD, the data in the literature support a two-stage mechanism. In particular, N-methyl-D-aspartate (NMDA) receptors seem to be hyperactive in early stages but rather hypoactive in later stages of the disease (e.g., Butterfield and Pocernich, 2003). Since NMDA receptors are heteromeric complexes of particular subunits and the subunit composition can be changed among others under pathological conditions (Cull-Candy et al., 2001), we decided to evaluate the Samaritan rat model via the expression of the NR1, NR2A and NR2B subunits of NMDA receptors. Although a gradual loss of cholinergic neurons in the nucleus basalis of Meynert reflects a gradual deterioration of memory and cognitive processes in AD (Arendt et al., 1983), changes in the activity of hippocampal or cortical presynaptic cholinergic nerve terminals also suggest possible two-stage mechanism. This activity can be estimated via measurements of Na^+^-dependent, high-affinity choline uptake (HACU) operating via CHT1 transporters. The HACU levels are increased in early stages of AD, probably via a compensatory reaction to impairments of the cholinergic basal forebrain system (Slotkin et al., 1990). On the contrary, the activity of CHT1 is markedly decreased in later stages of AD (Sims et al., 1983). Similarly, the number of membrane-bound CHT1 transporters estimated by means of the specific binding of [3H]hemicholinium-3 ([3H]HC-3), a selective and competitive inhibitor of HACU, is initially enhanced (Slotkin et al., 1990) but later attenuated (Pascual et al., 1991; Rodríguez-Puertas et al., 1994).

#### Tissue Sampling

Rats were sacrificed by cervical dislocation, decapitated and the brains rapidly removed. The frontal cortices and hippocampi, separately from the right (R) and left (L) hemisphere, were dissected and weighed. The frontal cortices were packed in aluminum foil and frozen at −40°C until assayed (no more than 2 weeks later), while the hippocampi were immediately used for preparation of synaptosomes.

#### Expressions of the NMDA Receptor Subunits NR1, NR2A and NR2B by Western Blotting

The frontal cortices were homogenized in 1.0 mL of lysis buffer (320 mM sucrose; 10 mM Tris, pH 7.4; 0.2 mM EDTA; 2 mM PMSF; 1 mM 2-mercaptoethanol and a cocktail of protease inhibitors, Sigma). Crude synaptosomal (P_2_) fractions were isolated from homogenates and resuspended in a loading buffer (63 mM Tris; 10% glycerol; 2% SDS; 5% 2-mercaptoethanol and 0.01% bromophenol blue). The protein concentration was determined by the Bradford method using bovine serum albumin (BSA) as the standard (Bio-Rad, CA, USA). The resuspended material was subjected to electrophoresis in a 7.5% polyacrylamide gel (Criterion Cell, Bio-Rad, CA, USA), followed by electroblotting in Criterion blotter (Bio-Rad, USA). Non-specific binding was blocked with 3% BSA dissolved in TBS-T buffer. Blots were incubated overnight with anti-NMDAR1 (1:100; Millipore, MA, USA) or for 2 h with anti-NMDAR2A/2B (1:500; Millipore, MA, USA) primary antibodies. For loading control, blots were treated with an anti-α-tubulin antibody (1:1000; Exbio, CZ, USA) for 1 h. Then, the blots were washed in TBS-T buffer and incubated for 1 h with a horseradish peroxidase-conjugated secondary antibody (1:3000; Dako, Denmark). Detections were performed with a chemiluminescent substrate (Pierce, WI, USA) and evaluated by the Gel Doc Analysis system (Bio-Rad, CA, USA).

#### Preparation of Hippocampal Synaptosomes

The hippocampi from individual animals were separately transferred to 0.32 M sucrose and immediately used for preparation of synaptosomes using glass-Teflon Potter’s Braun homogenizer, 0.32 M sucrose, an Universal 32R centrifuge (1000 g for 10 min at 4°C) and a Beckman J2-HS centrifuge (twice 20,000 g for 20 min at 4°C) in accordance with our previous studies (Kristofikova et al., 2004, 2010).

#### HACU Measurements

Hundred microlitres (100 μl) of synaptosomes were added to 880 μl of Krebs-Ringer-HEPES-glucose buffer (128 mM NaCl, 5 mM KCl, 2.7 mM CaCl_2_, 1.2 mM MgSO_4_, 5 mM glucose and 10 mM HEPES, pH = 7.4) and incubated for 4 min at 37°C with 20 μl of [3H]choline ([methyl-3H]choline chloride, PerkinElmer). The final concentration of the radioisotope was 10 nM and that of total proteins (estimated by the Bradford method with BSA as a standard) 150 μg/ml in all incubation mixtures. The incubation was terminated by rapid cooling and filtration under vacuum (Whatman BF/B filters). HACU was defined by its sensitivity to unlabeled HC-3 (RBI) and calculated as the difference between the uptake in samples incubated without and with 1 μM HC-3. The activity of samples was measured on a multi-purpose scintillation counter LS 6500 (Beckman Coulter) using Gold Star liquid scintillation cocktail (Meridian). Remaining synaptosomes were stored at −40°C and later used to measure the specific binding of [3H]HC-3.

#### Measurements of the Specific Binding of [3H]HC-3

Twenty microlitres (20 μl) of synaptic membranes were added to 170 μl of glycylglycine buffer (50 mM glycylglycine, 200 mM NaCl, pH = 7.8) and incubated for 30 min at room temperature with 10 μl of [3H]HC-3 ([methyl-3H]hemicholinium-3, diacetate salt, PerkinElmer). The final concentration of the radioisotope was 20 nM and that of total proteins was 150 μg/ml in all incubation mixtures. Parallel incubations in the presence of 10 μM HC-3 were performed to define the nonspecific binding. The titration and activity measurement were performed as described above.

#### Measured Parameters and Statistical Evaluations

In the AAPA task, total distance traveled within a session served as a measure of locomotor activity. Maximum avoidance time per session (s) and number of errors (entrances into the to-be-avoided sector) served as measures of cumulative within-session performance. Finally, latency to the first entrance from the beginning of the session (time to the first error) served as a measure of between-session learning. For the MWM we used total distance to reach the platform as a measure of acquisition performance in all sessions except in the probe trial. In the probe trials, where no platform was present, we evaluated preference for the quadrant that previously contained the platform.

Behavioral data from AAPA were analyzed separately for acquisition and reversal by two-way analysis of variance (ANOVA), with repeated measures on sessions and groups as a between subject factor. In cases of non-normality of the data (errors, maximum avoidance time and time to the first error) the natural logarithm (*ln*) was used to normalize the data distribution. *Post hoc* analysis was conducted with a Newman-Keuls test. Data from the hidden platform testing in the MWM were analyzed by mixed effects ANOVA, with repeated factors of swims and sessions, and groups as a between-subject factor. Data from the visible platform testing were analyzed by a two-way ANOVA (groups × swims) with repeated measures on swims. Data from the probe trial were analyzed with a two-sample, two tailed *t*-test. In all behavioral tests, 8 Samaritan rats and 10 controls were used. Missing values (i.e., tracks lost due to technical errors) were treated by a case-wise deletion; therefore, the degrees of freedom may differ for respective measures.

Biochemical data were analyzed by ANOVA with repeated measures with groups as a grouping factor and laterality (differences between the R and L side) as a within factor, and subsequently by one-way ANOVA. Differences between the R and L side were also characterized by the index of laterality [(L−R)/(L+R)]. This index is limited to zero when all the values are not lateralized (marked asymmetry was defined in this study by indexes of laterality > ± 0.090) or when the numbers of markedly R/L animals (dominance of the R side) and L/R animals (dominance of the L side) are approximately equal. Data are presented as the mean ± standard errors of the mean (SEM).

We also performed correlations between selected parameters of neurochemical tests. We correlated the expressions of NR1/NR2A/NR2B subunits of NMDA receptors, and CHT1 parameters (HACU and the specific binding of [3H]HC-3). For the sake of simplicity and straightforwardness, we report only significant correlations, and an absence of correlation is reported only if it has biological significance, using Pearson’s correlation (*r* and *p* values). The equality of correlation coefficients in two groups was examined using the test based on Fisher’s *Z*-transformation (*Z*-test). Significance was accepted in all cases of *p* < 0.05.

## Results

### Morris Water Maze

**Figure 1:**
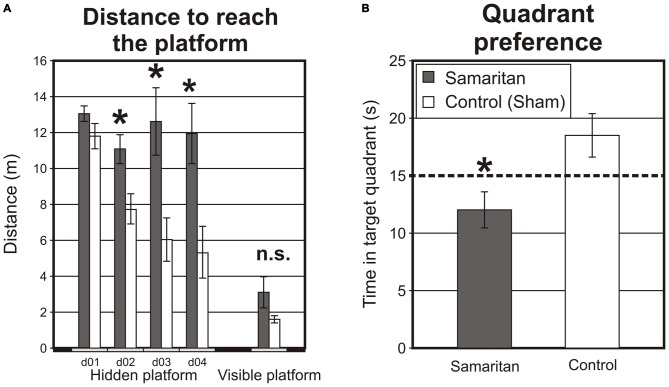
**Results from the Morris water maze (MWM). (A)** Total distance to reach the platform. In four daily acquisition sessions with a hidden platform in a stable position (d01–d04), Samaritan rats were significantly impaired and their performance did not improve during subsequent sessions, suggesting severe behavioral impairments. The visible platform test showed that both groups had both the motivation and physical ability to reach the platform (the apparent trend towards poorer performance in the Samaritan group was not statistically significant). **(B)** The probe trial (a single swim without a platform) was performed on the fifth session, after hidden platform training but before visible platform testing. Samaritan rats spent significantly less time in the quadrant that had previously contained the platform, which suggests an impairment of memory or spatial navigation. Their performance was even below the expected random value (15 s), as indicated by the dashed line. Columns in the charts show group means, and SEM is indicated by the error bars. Significant differences between groups or sessions at *p* < 0.05 as evaluated by a *t*-test on session-averaged data are indicated by an asterisk (*), n.s. indicates a non-significant result.

First we tested rats in the MMW (Figure 1). There were four acquisition sessions in the hidden platform version of the MWM with the platform located in the NE and pseudorandom starting positions. In these sessions, Samaritan rats needed longer paths to locate the platform, indicating that they were impaired. The mixed effects ANOVA (groups × sessions × swims) revealed a significant main effect of group (*F*_(1,13)_ = 12.62; *p* < 0.05), sessions (*F*_(3,39)_ = 4.76; *p* < 0.05), swims (*F*_(5,65)_ = 10.57; *p* < 0.05) and interaction between groups and session (*F*_(3,39)_ = 4.41; *p* < 0.05). No other interactions, including a triple interaction between swims, sessions and groups, were detected. The total distance to reach the platform in a particular session is illustrated in Figure 1A-left; though individual swims are not depicted, the total distance decreased in both groups for subsequent swims each day.

When examining the latency to reach the platform, the results were similar. The mixed effects ANOVA (groups × sessions × swims) revealed a significant main effect of group (*F*_(1,16)_ = 4.93; *p* < 0.05), sessions (*F*_(3,48)_ = 13.28 *p* < 0.05), swims (*F*_(5,80)_ = 4.47; *p* < 0.05) and interaction between groups and swims (*F*_(5,80)_ = 2.63; *p* < 0.05). No other interactions, including a triple interaction between swims, sessions and groups, were detected.

Second, a probe trial was conducted during session five of the MWM. A two-sample *t*-test comparing the performance between groups revealed decreased time spent in the target quadrant that previously contained the platform (*T*_(1,16)_ = 10.13; *p* < 0.05). As can be seen from Figure 1B, the performance of control rats was above the level of chance (15 s for a randomly swimming rat), but the performance of Samaritan rats was below chance. This suggests that remembrance of the platform position was impaired in the Samaritan group. (Figure 1B) The low target sector preference of the Samaritan group in the probe trial was probably linked to their inferior searching strategy. Visual inspection of the data suggested much higher thigmotaxis in the Samaritan group, and a slight preference for the quadrant opposite to the target (where the starting position was located). We thus evaluated thigmotaxis (measured as the distance from the center of the maze) by a two-sample *t*-test, which confirmed that the Samaritan rats tended to spend more time on the periphery (*T*_(1,16)_ = 2.78; *p* < 0.05).

Finally, we conducted a visible platform test (Figure 1A-right, showing the mean path needed to find the platform for each day), consisting of eight swims in 1 day. The two-way ANOVA (groups × swims) failed to detect a significant main effect of group (*F*_(1,14)_ = 3.44; *p* > 0.05), but a significant main effect of swims was found (*F*_(7,98)_ = 3.21; *p* < 0.05). Visual inspection of the data indicated poorer performance in the initial swims (not shown), before the animals adjusted to the new task rules; however, there was no significant interaction (*F*_(7,98)_ = 1.65; *p* > 0.05). For the time to reach the platform, there was no significant effect of group (*F*_(1,16)_ = 3.93; *p* > 0.05), but there was a significant effect of swims (*F*_(7,112)_ = 5.37; *p* < 0.05. Again, no significant interaction was present (*F*_(7,112)_ = 2.06; *p* > 0.05).

### Active Allothetic Place Avoidance

#### Habituation Sessions

**Figure 2:**
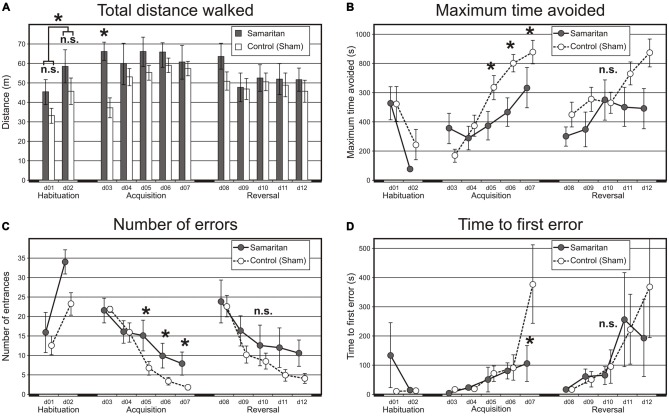
**Results from the Carousel maze.** The Carousel maze testing consisted of two habituation sessions (d01–02), acquisition (d03–07) and reversal training (d08–12). **(A)** Total distance walked is a measure of locomotor activity. In the habituation sessions, the difference between groups was not significant, but locomotion increased in the second session (with a rotating arena) relative to the first (with a stable arena). In the acquisition phase, the effect of group was also not significant; however, the control group gradually increased its locomotion, whereas the locomotion of the Samaritan rats was stable. In the reversal stage, no difference was found. **(B)** Maximum avoidance time indicates the longest period of successful avoidance during a session. Again, the effect of groups was not significant, but there was a significant interaction suggesting slower learning in the Samaritan group in the acquisition phase. In the reversal phase, neither the group effect nor interaction was significant, despite the apparent trend visible in the graph. **(C)** Number of errors (entrances into the sector) is another measure of avoidance behavior. The difference between groups was again not significant, but a significant interaction indicated slower learning and poorer final performance in Samaritans during the acquisition phase. In reversal, the apparent tendency toward increased number of errors in the Samaritan group remained non-significant. **(D)** Time to first error is a measure of between-session (long-term) memory. There was only a trend for group difference, but a significant interaction term again indicated poorer final performance in Samaritans during acquisition. There was no significant difference in the reversal phase, which may have been caused by the large variance in this parameter. Bars or data points in the charts show group means, and SEM is indicated by the error bars. Significant differences between groups at *p* < 0.05 as evaluated by the *t*-test on session-averaged data are indicated by an asterisk (*), n.s. indicates a non-significant result.

During the habituation phase (two sessions; Figure 2-left parts of panels), no effect of groups was detected (*F*_(1,15)_ = 2.35; *p* > 0.05). There was, however, a significant effect of days, with animals walking more on the second day of habituation on the rotating arena (*F*_(1,15)_ = 4.74; *p* < 0.05). No interaction between the factors of group and day was detected. Place navigation parameters were not evaluated in this phase, since there were no shocks applied and therefore no avoidance.

#### Acquisition Sessions

During the acquisition phase, there was no effect of groups on total distance (*F*_(1,16)_ = 1.84; *p* > 0.05); however, ANOVA revealed a significant effect of sessions (*F*_(4,64)_ = 3.09; *p* < 0.05) and a significant interaction (*F*_(4,64)_ = 4.43; *p* < 0.05). The Newman-Keuls *post hoc* test on session term revealed that the distance was higher in the last three sessions, probably as an adaptation to the task. Analysis of the interaction term demonstrated that while Samaritan rats had stable locomotion in all acquisition sessions, the control group gradually increased the total distance traveled (Figure 2A). Visual inspection of the data showed that some control animals exhibited passive behavior during the first avoidance session, but they gradually abandoned this strategy, and all of them were able to achieve successful avoidance. In the Samaritan group, this passive behavior was less common in the beginning, but several rats were not able to achieve the avoiding behavior at all, although some others were.

Analysis of the maximum avoidance time again revealed a significant main effect of sessions (*F*_(4,64)_ = 16.19; *p* < 0.05) but not groups (*F*_(1,16)_ = 1.79; *p* > 0.05), with the interaction term being significant (*F*_(4,64)_ = 5.70; *p* < 0.05). *Post hoc* test on sessions revealed that again, maximum avoidance time was significantly increased in last three acquisition sessions (*p* < 0.05). Analysis of the interaction term revealed that Samaritan rats had lower maximum avoidance times at the end of acquisition than controls (*p* < 0.05; Figure 2B).

Analysis of the number of errors revealed a significant main effect of sessions (*F*_(4,56)_ = 30.90; *p* < 0, 05), but not group (*F*_(1,14)_ = 2.93; *p* > 0.05]; however, the interaction term was significant (*F*_(4,56)_ = 3.02; *p* < 0.05. The *post hoc* test on the session factor revealed a lower number of errors in last three sessions compared to the first two sessions (*p* < 0.05). Analysis of the interaction term showed that Samaritan rats did not achieve the same level of final performance as controls (*p* < 0.05; Figure 2C).

The two-way ANOVA conducted on the data for time-to-the-first-error revealed a significant effect of sessions (*F*_(4,64)_ = 9.85; *p* < 0.05) and the interaction term (*F*_(4,64)_ = 3.26; *p* < 0.05). The effect of group exhibited only a trend (*F*_(1,16)_ = 3.98; *p* = 0.06), likely due to the high variance in the data. The* post hoc* test of the session factor showed that rats in the last two sessions had increased time to the first error compared to the three initial sessions. Visual analysis of the group factor trend showed that control groups improved in between-session memory compared to Samaritan rats, although there was high variation. The *post hoc* analysis of the interaction term showed impaired between-session memory in Samaritan rats compared to controls (Figure 2D).

All these data suggest that the AD model animals were impaired in acquisition sessions for all measured spatial parameters, including the total distance (significant interaction term).

#### Reversal Sessions

Total distance in the reversal sessions was not affected by any factor, nor was any interaction detected (all *p* > 0.05). This suggests that animals were already accommodated to the task and did not have locomotor disabilities (Figure 2A-right). The two-way ANOVA conducted on maximum avoidance time revealed only a main effect of sessions (*F*_(4,60)_ = 6.17; *p* < 0.05). No other terms (groups, interaction) were significant (all *p*s > 0.05). This again suggests that there were no between group differences in this parameters in the reversal sessions, although a visual trend of an increased number of errors in Samaritan rats can be seen (Figure 2B-right). The number of errors was affected only by sessions (*F*_(4,44)_ = 14.59; *p* < 0.05); no other effects were significant (all *p*s > 0.05). The *post hoc* analysis of the session factor showed that in the first reversal session this parameter was worse than in subsequent sessions, when it gradually decreased (Figure 2C-right). There seems to be a trend in interaction, but this was not significant, probably due to high variation. Time to the first error was not affected by any factor, and this parameter also showed high variation (all *p*s > 0.05; Figure 2D-right).

### Neurochemical Analysis

**Figure 3:**
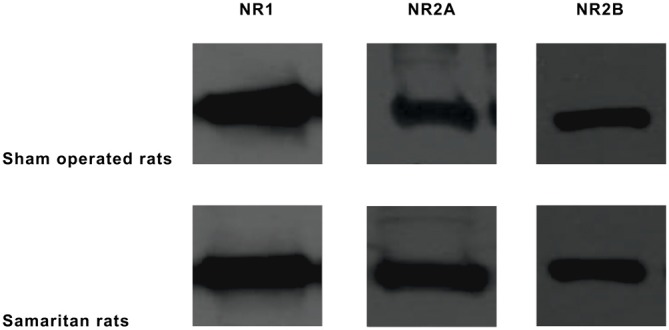
**Results of western blotting.** Representative images of samples from the L hemisphere were used (all data are presented in Table 2).

Table 2 shows the expression levels of NR1/NR2A/NR2B in the R and L frontal cortices. For representative image of western blot results, see Figure 3. In the sham-operated Long Evans controls, no marked asymmetries were found in NR1 (index of laterality = +0.005) or in NR2A subunit (index of laterality = −0.010). On the other hand, mild L/R dominance was observed in the NR2B subunit (index of laterality = +0.056, there was an increase to 112% in the L compared to the R cortex, and the results of ANOVA with repeated measures for laterality was significant, *p* < 0.001). In Samaritan rats compared to the controls, results of ANOVA with repeated measures and one-way ANOVA only showed a significant change in the NR2A subunit (an increase to 102% in the L side of Samaritan rats). Although the results of the global test also suggested possible effects of laterality in the NR1 and NR2B subunits (with a drop to 98% of NR1 and an increase to 104% of NR2B in Samaritan rats, in both cases in the R side), results of one-way ANOVA did not support this.

**Table 2:** **Expression of NR1/NR2A/NR2B subunits of NMDA receptors in the frontal cortex**.

Groups	*n*	R	L	L−R/L+R
**NR1**
Sham-operated rats	10	1.002 ± 0.006	1.009 ± 0.004	0.005 ± 0.003
Samaritan rats	8	0.985 ± 0.014	1.009 ± 0.004	0.013 ± 0.007
One-way ANOVA		*F*_(1,16)_ = 1.29,	*F*_(1,16)_ = 0.00,	*F*_(1,16)_ = 1.11,
		*p* = 0.2728	*p* = 0.9906	*p* = 0.3068
**NR2A**
Sham-operated rats	10	0.856 ± 0.004	0.840 ± 0.003	−0.010 ± 0.003
Samaritan rats	8	0.864 ± 0.006	0.857 ± 0.005*	−0.005 ± 0.004
One-way ANOVA		*F*_(1,16)_ = 1.46,	*F*_(1,16)_ = 7.56,	*F*_(1,16)_ = 0.91,
		*p* = 0.2439	*p* = 0.0142	*p* = 0.3548
**NR2B**
Sham-operated rats	10	0.850 ± 0.020	0.948 ± 0.007	0.056 ± 0.015
Samaritan rats	8	0.883 ± 0.009	0.950 ± 0.007	0.036 ± 0.008
One-way ANOVA		*F*_(1,16)_ = 1.91,	*F*_(1,16)_ = 0.03,	*F*_(1,16)_ = 1.16,
		*p* = 0.1863	*p* = 0.8540	*p* = 0.2969

Mean ± SEM. Expressions of NMDA receptor subunits were estimated separately in the right (R) and left (L) frontal cortices. Results of ANOVA with repeated measures: NR1 groups: F_(1,16)_ = 0.93, p = 0.3499, laterality: F_(1,16)_ = 5.22, p = 0.0363*, interaction: F_(1,16)_ = 1.45, p = 0.2455. NR2A groups: F_(1,16)_ = 6.43, p = 0.0220*, laterality: F_(1,16)_ = 7.89, p = 0.0126*, interaction: F_(1,16)_ = 0.96, p = 0.3421. NR2B groups: F_(1,16)_ = 2.85, p = 0.1107, laterality: F_(1,16)_ = 28.47, p = 0.0001***, interaction: F_(1,16)_ = 1.04, p = 0.3220. *p < 0.050, ***p < 0.001.

Table 3 demonstrates the results of the HACU and [3H]HC-3 specific binding measurements in the R and L hippocampi. In sham-operated Long Evans controls, the data indicated a marked R/L dominance of HACU activity (index of laterality −0.094, an increase approximately to 119% in the R compared to the L hippocampus, and significant ANOVA with repeated measures for laterality *p* < 0.05). On the other hand, R/L dominance was not found in [3H]HC-3 specific binding (index of laterality -0.027, and an insignificant increase to 108% in the R compared to L side). When comparing Samaritan rats to controls, results of ANOVA with repeated measures indicated significant differences between groups in HACU values (increases to 142% in the R side and to 132% in the L side of Samaritan rats); nevertheless, the results of one-way ANOVA were only borderline significant.

**Table 3:** **Activity and number of synaptic CHT1 transporters in the hippocampus**.

Groups	*n*	R	L	L−R/L+R
**HACU**
Sham-operated rats	9	226.2 ± 24.0	189.7 ± 24.4	−0.094 ± 0.057
Samaritan rats	9	321.1 ± 38.6	251.1 ± 27.9	−0.114 ± 0.064
One-way ANOVA		*F*_(1,16)_ = 4.36,	*F*_(1,16)_ = 2.74,	*F*_(1,16)_ = 0.05,
		*p* = 0.0532	*p* = 0.1174	*p* = 0.8191
**[3H]HC-3**
Sham-operated rats	9	4465.1 ± 674.3	4123.5 ± 466.5	−0.027 ± 0.033
Samaritan rats	9	4520.3 ± 540.8	4532.5 ± 333.4	0.020 ± 0.048
One-way ANOVA		*F*_(1,16)_ = 0.00,	*F*_(1,16)_ = 0.51,	*F*_(1,16)_ = 0.62,
		*p* = 0.9499	*p* = 0.4859	*p* = 0.4408

Mean ± SEM. The activity of the high-affinity choline uptake (HACU) was estimated in synaptosomes isolated separately from the right (R) and left (L) hippocampi and expressed as fmoles/4 min/mg or proteins. The specific binding of (3H)HC-3 was estimated in hippocampal synaptic membranes and expressed as fmoles/mg of proteins. Results of ANOVA with repeated measures: HACU groups: F_(1,16)_ = 5.15, p = 0.0374*, laterality: F_(1,16)_ = 5.30, p = 0.0351*, interaction: F_(1,16)_ = 0.53, p = 0.4791. [3H]HC:3 binding—groups: F_(1,16)_ = 0.11, p = 0.7399, laterality: F_(1,16)_ = 0.41, p = 0.5307, interaction: F_(1,16)_ = 0.47, p = 0.5007. *p < 0.050.

No differences between groups were observed in the specific binding of [3H]HC-3.

### Correlation Analysis

The results of correlation analysis from biochemical experiments are summarized in Table 4. In sham-operated controls, there were significant relationships between all subunit expression levels in the R side and their laterality (three markedly negative correlations), between NR2B expression in the L side and its laterality (positive correlation), and finally between particular subunits with respect to their laterality (between NR1 and NR2B or between NR2A and NR2B). A comparison of controls and Samaritan rats indicated two significant differences (a shift from a positive to a negative correlation between the laterality of NR1 and NR2B in the R side, a shift from negative to a markedly positive correlation between the laterality of NR1 and the laterality of NR2B). With respect to CHT1, correlation analysis revealed two marked positive correlations between the R and L side in the controls (for HACU as well as [3H]HC-3 binding); however, no differences between control and Samaritan rats were found. Moreover, correlation analysis revealed links between some subunits and CHT1 in sham-operated controls (two positive correlations, between NR2A in the R side and HACU laterality and between the laterality of NR2B and HACU in the R side; two negative correlations, between NR2B and HACU, both in the R side, and between NR2B in the L side and [3H]HC-3 in the R side. A comparison of control and Samaritan rats indicated three significant differences (a shift from a negative to a positive correlation between NR1 and HACU both in the L hemisphere, an increased positive correlation between the laterality of NR1 and that of [3H]HC-3, and finally a shift from a negative to a positive correlation between NR2A in the R side and [3H]HC-3 in the L side).

**Table 4:** **Correlation analysis of data from biochemical experiments**.

	Sham-operated		Samaritan	
Parameter vs. parameter	*r*	*p*	*r*	*p*	*Z*-test *p*
Subunits of NMDA receptors
NR1 R vs. Laterality NR1	−0.789	0.007**	−0.976	< 0.001***	0.052
Laterality NR1vs. NR2B R	0.356	0.312	−0.692	0.057	0.037*
Laterality NR1 vs.	−0.245	0.495	0.72	0.044*	0.048*
Laterality NR2B
NR2A R vs. Laterality NR2A	−0.719	0.019*	−0.702	0.052	0.953
NR2A L vs. Laterality NR2B	−0.719	0.048*	−0.86	0.006**	0.356
NR2B R vs. Laterality NR2B	−0.979	< 0.001***	−0.94	< 0.001***	0.361
NR2B L vs. Laterality NR2B	0.665	0.036*	0.878	0.004**	0.334
CHT1 transporters
HACU R vs. HACU L	0.686	0.041*	0.105	0.805	0.225
	0.903	< 0.001***	0.676	0.065	0.271
NMDA subunits and CHT1
NR1 L vs. HACU L	−0.6	0.088	0.595	0.119	0.023*
Laterality NR1 vs.	0.438	0.238	0.935	< 0.001***	0.043*
Laterality [3H]HC-3
NR2A R vs. Laterality HACU	0.711	0.032*	0.159	0.707	0.229
NR2A R vs. [3H]HC-3 L	−0.584	0.099	0.488	0.22	0.047*
NR2B R vs. HACU R	−0.74	0.023*	0.151	0.757	0.074
NR2B L vs. [3H]HC-3 R	−0.705	0.034*	−0.473	0.236	0.549
Laterality NR2B vs. HACU R	0.684	0.042*	−0.276	0.509	0.064

The laterality of particular parameters was expressed as indexes of laterality. Correlation coefficients (r) were evaluated via a test based on Fisher’s Z-transformation (Z-test). *p < 0.050, **p < 0.010, ***p < 0.001.

## Discussion

### Impairments in the Morris Water Maze

Our results show that Samaritan rats were impaired in the reference memory version of the MWM. They took longer distances and times to reach the platform, and impairment was also present in the probe trial, where the experimental group had a significantly lower preference for the target quadrant that had previously contained the platform. In the visible platform testing, no significant difference between the groups was found.

Memory impairments in the MWM are among the major behavioral hallmarks of rodent models of AD. This task is a generally recognized model of AD because it taps hippocampal functions, which are among the first affected in human AD, is very simple and is widely used in existing studies, making it advantageous for comparisons of different models (for review, see D’Hooge and De Deyn, 2001; Sabbagh et al., 2013).

Although Lecanu et al. (2006) also examined MWM performance in the Samaritan model, they only tested the effects of experimental manipulations on the retrieval of memories acquired prior to the surgery. Our experiments assessed the ability to learn the reference memory task in rats already affected by the AD model, as well as the ability (and accuracy) of retrieval in a probe trial and the capability of the rats to master the procedural aspects of the task.

Trials using a visible platform should assess the spatial memory-independent aspects of the task, e.g., the capability to swim, ability to perceive visual cues, procedural learning and motivation to reach the platform. Although Figure 1A may suggest a trend toward poorer performance in Samaritan rats, the difference between the groups was not significant. It is also possible that the animals partly relied on a spatial strategy even during the visible-platform test, as we did not change the visible platform position across swims. Because swimming speed was not altered in the Samaritan group (not shown), we can rule out potential locomotor impairment in the AD model.

### Deficits in Active Allothetic Place Avoidance

Compared to the control group, we also observed a significant deficit of Samaritan rats in the acquisition phase of the active place avoidance test. No differences between groups were detected in the habituation phase, when only distance was evaluated (as there was no to-be-avoided sector). The lack of differences between total distances in the habituation phase (analogous to open field testing) suggests that spontaneous locomotion in Samaritan rats is normal, and confirms that no gross motoric impairments are present.

The deficits we found in the acquisition sessions (demanding mainly so-called cognitive coordination), with Samaritan rats showing a learning impairment, are a completely novel finding. There was a clear difference between groups in all spatial parameters (detected as significant interaction terms). The significant interaction term found in the total distance in acquisition sessions shows that control animals gradually increased their distance, as they adopted an active strategy enabling them to solve the task. The Samaritan rats exhibited stable (and quite high) locomotion on all days, but they nevertheless failed to reach the level of avoidance seen in the control group.

We hypothesized that a deficit in cognitive coordination would be present in the Samaritan rat model, because a meta-analysis of the Stroop effect showed impaired processing of multiple information streams in human AD patients (Ben-David et al., 2014). The deficit we observed in active place avoidance could be caused by a disruption of cognitive coordination; however, a general impairment of spatial navigation and memory (as also observed in the MWM) is in itself sufficient to explain the decreased performance in this task. Unfortunately, there are no other studies on animal models of AD and their possible deficits in cognitive coordination, so this question remains open for future studies.

In the reversal sessions of active place avoidance, demanding cognitive flexibility, we observed no significant differences between groups. Here the situation is much more difficult to interpret. From Figures 2B,C it is apparent that the performance of the Samaritan group actually continued to be worse even in the reversal sessions, although the difference failed to reach statistical significance, probably because the reversal performance was more variable. This variability could be related to higher stress levels and/or elevated cognitive demands. It is conceivable that the change of sector position was relatively more disturbing for the controls, which better remembered the original sector position, than for the Samaritans, and this reduced the apparent differences between them. Other observations from our laboratory (Hatalova et al., 2014) may support such an explanation.

### Alterations in the Cortical Glutamatergic and Hippocampal Cholinergic Systems

In the present study, we show that there are asymmetrical differences in cortical NR2B subunit expression levels and in the activity, rather than the number, of hippocampal CHT1 transporters in adult male Long Evans controls (compare Tables 2, 3). Links between particular subunits of the NMDA receptor or between the R and L sides in the case of subunits/CHT1 could also be supported by our correlation analyses (Table 4); however, these results should not be over-interpreted since our experiments were not performed on intact animals. Nevertheless, both results are in accordance with observed lateral differences in hippocampal NR2B subunit expression in mature mice (Kawakami et al., 2003) and in hippocampal HACU in adult Long Evans rats (Kristofikova et al., 2004, 2010), and so support the hypothesis that changes in NMDA receptors and CHT1 transporters could be a molecular basis for the structural and functional asymmetry of the mature brain (Gibbs, 2000; Kawakami et al., 2003; Kristofikova et al., 2004).

A comparison of Samaritan rats with controls revealed rather unilateral changes in the frontal cortex (significantly increased NR2A expression in the L side, see Table 2) but bilateral alterations in the hippocampus (significantly increased HACU levels in both hemispheres, see Table 3). Nevertheless, the significant result of ANOVA with repeated measures for the laterality of NR1 does not exclude moderate alterations in NR1 expression in the R side of the frontal cortex (in contrast to NR2B, the effect of laterality in NR1 cannot be associated with the asymmetry of this subunit in the controls). These results may be also supported by significant correlation differences between NR1 and NR2B (Table 4).

Since the NR2A subunit becomes more prevalent in adulthood and with advanced aging (Cui et al., 2013) but is markedly attenuated in the autoptic cortical or hippocampal regions of people with AD (e.g., Hynd et al., 2004), our results of increased NR2A expression in the frontal cortex of Samaritan rats could be thus interpreted via the animal model mimicking early rather than terminal stages of AD. Moreover, with respect to the hyperactivity/hypoactivity of the glutamatergic system observed in AD (Butterfield and Pocernich, 2003), the increased NR2A subunit expression could reflect rather its hyperactivity since NR2A-containing receptors have a lower activation energy compared to those with NR2B (Erreger et al., 2005). We also detected bilateral increases in the activity of CHT1 in Samaritan rats compared to controls. Similar changes in CHT1 transporters observed in Samaritan rats and in people in early stages of AD (Slotkin et al., 1990) also support our above-mentioned hypothesis that Samaritan rats could be a promising model of sporadic AD, especially in its early stage.

### Relationships Between Cortical NMDA Receptors and Hippocampal CHT1

Our correlation analysis suggests complicated links between the expression of NMDA receptors in the frontal cortex and hippocampal CHT1 in the controls (Table 4). In particular, the data indicate possible connections between NR2A/NR2B and CHT1 activity as well as between NR2B and the number of CHT1; however, a more detailed analysis should be performed in the future to correctly interpret this hypothesis. Nevertheless, our results agree well with the reported role of prefrontal-hippocampal pathways in cognitive/memory processes, and perhaps also with the role of the R prefrontal cortex observed in human- or non-human primates (Anderson et al., 2015). On the other hand, an interpretation of changes in Samaritan rats compared to controls is not difficult. Namely, higher positive correlations between NR1 and CHT1 probably reflect similar changes in the glutamatergic and cholinergic systems mediated by applications of Abeta_42_ and the two pro-oxidative substances into the L cerebral ventricle.

### Study Limitations and Caveats

Despite what we feel are significant advances, our study is not without limitations: First, we did not measure any classical signs of AD pathology, such as amyloid plaques, neurofibrillary tangles etc. Rather we strongly relied on the published results of Lecanu et al. (2006) and the patented Samaritan model, and we used already-operated animals. Second, we found a visual trend to a worsening in performance in the visible platform version of the MWM in Samaritan rats, which might suggest some deficit in procedural aspects of the task (e.g., decreased visual acuity or motivation), but we argue that such a deficit only partially contributed to the deficit in the hidden platform task. This interpretation is also supported by the fact that in the active place avoidance task, which is procedurally very different and less dependent on precise visual navigation, the cognitive deficit of Samaritan rats was robust and manifested in multiple parameters.

The Samaritan model itself is limited by the fact that it exhibits only face validity (AD-like symptoms), but not construct validity (similarities in etiology), as it is induced by the artificial application of Abeta together with supplementary chemicals, which is obviously not the case in actual AD. Furthermore, the unilateral injection of the solution limits the validity of the model with regard to lateralized changes in neurotransmitter systems. However, as the etiology of sporadic AD remains enigmatic, most of the existing models focus either on the familial form of the disease, or must deal with similar issues.

## Conclusion

In summary, our results corroborate the working hypothesis that the Samaritan rat model of AD presents with deficits in both behavioral parameters tested with two spatial tasks, as well as alterations in the cortical glutamatergic and hippocampal cholinergic systems. The deficit in cognitive functions in Samaritan rats and the changes in NR2A subunit expression and in CHT1 transporters support the notion that Samaritan rats are a promising animal model of early-stage AD manifesting in changes in behavior even in young adult rats, and a candidate model of the sporadic form of the disease.

## Author Contributions

TP contributed to the experimental design, performed behavioral experiments including analysis and wrote part of the manuscript; MS, KM and IV performed part of the behavioral experiments. ZK conceived, performed and analyzed neurochemical studies and participated significantly in manuscript writing, HM and JS performed part of the neurochemical analyses. KV co-designed the experiment and provided practical organization of the study. DR co-designed the study and provided leadership for neurochemistry experiments, AS designed the study, wrote major parts of the manuscript, interpreted the data and provided scientific leadership.

## Conflict of Interest Statement

The authors declare that the research was conducted in the absence of any commercial or financial relationships that could be construed as a potential conflict of interest. The reviewer SCC and handling Editor declared their shared affiliation, and the handling Editor states that the process nevertheless met the standards of a fair and objective review.

## Abbreviations

AAPA: active allothetic place avoidance
Abeta: amyloid beta
AD: Alzheimer’s disease
CHT1: high-affinity choline transporter
HACU: high-affinity choline uptake
MWM: Morris water maze
NMDA: N-methyl-D-aspartate.
